# Supplementary material for: Sea cucumbers reduce chromophoric dissolved organic matter in aquaculture tanks
Source: PeerJ. 2018 Feb 5;6:e4344. doi: 10.7717/peerj.4344 (PMC5804323; doi:10.7717/peerj.4344)
Supplement: Supplemental Information 3 — CDOM and ancillary data in the inlet, tanks and effluents. [file peerj-06-4344-s003.docx]

**Table S1.** **CDOM and ancillary data of inlet water.** The a _325_ values are the absorption coefficients at wavelength 325 nm (m^-1^), the S_275-295_ values are the spectral slopes at the wavelength band from 275 to 295 nm (μm^-1^), and S_R_ is the ratio of the slope between 275 and 295 nm divided by the slope between 350 and 400 nm (unitless). TDS is total dissolved solid, TOC is total organic carbon in mg C l^-1^, POM is particulate organic carbon in mgl^-1^, chl *a* is the concentration of chlorophyll a in μg l^-1^ and BA is the bacterial abundance in (x10^6^ cells ml^-1^). x-no datum

| Date  (dd/mm/yy) | pH | Temp  (ºC) | Salinity  (psu) | TDS | a_325_  (m^-1^) | S_275-295_  (μm^-1^) | S_R_ | TOC  (mg C l^-1^) | POM  (mg l^-1^) | Chlorophyll *a*  (μg l^-1^) | Bacteria abundance  (x 10^6^ cells ml^-1^) |
| --- | --- | --- | --- | --- | --- | --- | --- | --- | --- | --- | --- |
| 17/07/13 | 8.16 | 21.02 | 35.7 | 26.55 | 0.38 | 20.0 | 1.85 | 0.11 | 5.07 | x | 9.76 |
| 30/07/13 | 8.18 | 21.58 | 35.91 | 27.14 | 0.70 | 19.0 | 1.31 | 0.35 | 3.85 | 1.25 | 18.94 |
| 29/08/13 | 8.09 | 25.58 | 37.48 | 28.21 | 0.43 | 20.0 | 2.05 | 0.23 | x | 0.42 | 8.19 |
| 13/09/13 | 8.11 | 23.74 | 38.8 | 29.05 | 0.83 | 16.3 | 1.52 | x | 3.21 | 0.72 | 5.98 |
| 27/09/13 | 8.06 | 23.2 | 35.8 | 20.27 | 0.20 | 26.7 | 1.89 | 0.12 | 2.98 | 0.29 | 5.55 |
| 15/10/13 | 8.15 | 19.86 | 37.68 | 18.26 | 0.50 | 18.7 | 1.35 | 0.14 | 2.40 | 0.46 | 6.52 |
| 30/10/13 | 8.15 | 16.78 | 37.45 | 28.14 | 0.46 | 18.2 | 1.40 | 0.13 | 2.51 | 0.27 | 4.24 |
| 13/11/13 | 8.15 | 17.28 | 37.93 | 28.48 | 0.25 | 23.5 | 1.93 | 0.19 | 2.31 | 0.57 | 0.50 |
| 02/12/13 | 8.27 | 16.29 | 39.92 | 29.75 | 0.24 | 20.5 | 2.63 | 0.21 | 2.33 | 0.29 | 4.13 |
| 17/12/13 | 8.31 | 16.28 | 38.11 | 28.56 | 0.18 | 28.0 | 2.15 | 0.36 | 2.31 | 0.97 | 4.79 |
| 30/12/13 | 8.22 | 14.29 | 41.54 | 30.84 | 0.21 | 24.1 | 1.94 | 0.37 | 1.20 | 0.74 | 20.74 |
| 15/01/14 | 8.23 | 14.8 | 41.39 | 30.79 | 0.20 | 24.3 | 1.76 | 0.28 | 1.20 | 0.87 | 1.64 |
| 30/01/14 | 8.05 | 13.87 | 38.25 | 28.7 | 0.45 | 15.2 | 1.21 | 0.09 | 0.60 | 2.35 | 2.33 |
| 11/02/14 | 7.87 | 13.58 | 41.65 | 30.94 | 0.18 | 22.8 | 2.04 | 0.09 | 1.28 | 1.07 | 1.58 |
| 27/02/14 | 7.71 | 14.31 | 41.3 | 30.71 | 0.06 | 19.3 | 2.05 | 0.31 | 1.39 | 0.41 | 3.82 |
| 14/03/14 | 7.89 | 15.2 | 40.47 | 30.14 | 0.08 | 24.0 | 1.63 | 0.43 | 0.39 | 0.28 | 14.29 |
| 27/03/14 | 7.9 | 14.37 | 38.24 | 28.64 | 0.06 | 24.3 | 1.76 | 0.13 | 1.08 | 0.50 | 3.17 |
| 11/04/14 | 8.04 | 17.18 | 36.44 | 27.43 | 0.07 | 38.1 | 0.64 | 0.17 | 1.75 | 0.34 | 2.78 |
| 30/04/14 | 7.97 | 18.03 | 38.25 | 28.65 | 0.07 | 24.7 | 1.79 | 0.17 | 6.27 | 2.62 | 4.22 |
| 14/05/14 | 7.79 | 19.2 | 37.47 | 28.13 | 0.14 | 10.3 | 0.63 | 0.26 | 2.25 | 0.37 | 2.93 |
| 29/05/14 | 7.94 | 16.41 | 37.2 | 27.96 | 0.40 | 19.0 | 1.44 | 0.18 | 1.80 | 0.48 | 5.68 |
| 11/06/14 | 8.05 | 18.94 | 36.96 | 27.78 | 0.36 | 21.6 | 1.58 | 0.09 | 2.47 | 0.57 | 4.83 |
| 30/06/14 | 7.56 | 17.17 | 36.22 | 27.28 | 0.36 | 19.7 | 1.59 | x | 1.60 | 0.33 | 7.39 |
| 11/07/14 | 8.1 | 17.96 | 38.22 | 20.62 | 0.58 | 18.5 | 1.25 | x | 2.45 | 0.66 | 10.24 |
| 25/07/14 | 7.98 | 19.82 | 36.95 | 27.78 | 0.18 | 27.9 | 1.22 | 0.17 | 2.50 | 0.70 | 8.96 |
| 20/08/14 | 8.06 | 25.04 | 38.88 | 29.15 | 0.31 | 23.1 | 1.22 | 0.13 | 2.45 | 0.84 | x |

**Table S2.** **CDOM and ancillary data of the *+holothurian* effluent water.** The a _325_ values are the absorption coefficients at wavelength 325 nm (m^-1^), the S_275-295_ values are the spectral slopes at the wavelength band from 275 to 295 nm (μm^-1^), and S_R_ is the ratio of the slope between 275 and 295 nm divided by the slope between 350 and 400 nm (unitless). TDS is total dissolved solid, TOC is total organic carbon in mg C l^-1^, POM is particulate organic carbon in mgl^-1^, chl *a* is the concentration of chlorophyll a in μgl^-1^ and BA is the bacterial abundance in (x10^6^ cells ml^-1^).

| Date  (dd/mm/yy) | pH | Temp  (ºC) | Salinity  (psu) | TDS | a_325_  (m^-1^) | S_275-295_  (μm^-1^) | S_R_ | TOC  (mgC l^-1^) | POM  (mg l^-1^) | Chlorophyll *a*  (μg l^-1^) | Bacteria abundance  (x10^6^ cells ml^-1^) |
| --- | --- | --- | --- | --- | --- | --- | --- | --- | --- | --- | --- |
| 17/07/13 | 8.18 | 21.04 | 35.71 | 26.96 | 0.38 | 15.2 | 1.57 | 0.12 | 4.59 | x | 1.09 |
| 30/07/13 | 8.19 | 21.48 | 35.86 | 27.7 | 0.59 | 13.6 | 1.32 | 0.20 | 3.87 | 1.04 | 20.24 |
| 29/08/13 | 8.13 | 25.29 | 37.39 | 28.15 | 0.31 | 18.0 | 2.07 | 0.20 | 4.49 | 0.62 | 8.13 |
| 13/09/13 | 8.14 | 23.84 | 38.72 | 28.55 | 0.45 | 15.7 | 1.99 | 0.09 | 3.30 | 0.12 | 4.77 |
| 27/09/13 | 8.09 | 22.8 | 35.89 | 21.03 | 0.41 | 16.7 | 1.82 | 0.15 | 4.17 | 0.32 | 8.41 |
| 15/10/13 | 8.2 | 19.73 | 37.57 | 28.17 | 0.53 | 13.1 | 1.32 | 0.15 | 2.13 | 0.52 | 7.93 |
| 30/10/13 | 8.15 | 17.28 | 37.46 | 28.12 | 0.34 | 16.2 | 2.03 | 0.16 | 3.86 | 0.20 | 4.33 |
| 13/11/13 | 8.14 | 16.69 | 38.16 | 28.6 | 0.32 | 14.5 | 2.34 | 0.18 | 2.59 | 1.01 | 5.94 |
| 02/12/13 | 7.93 | 12.36 | 40.36 | 30.15 | 0.32 | 12.2 | 3.13 | 0.26 | 2.01 | 0.48 | 13.96 |
| 17/12/13 | 8.2 | 14.51 | 37.67 | 28.29 | x | - | x | x | 2.24 | 0.37 | 10.34 |
| 30/12/13 | 7.87 | 14.07 | 41.67 | 30.96 | 0.13 | 17.2 | 2.07 | 0.29 | 1.70 | 0.32 | 7.23 |
| 15/01/14 | 8.23 | 14.76 | 41.59 | 30.89 | 0.21 | 22.5 | 2.59 | 0.32 | 1.60 | 2.48 | 3.92 |
| 30/01/14 | 8.04 | 12.77 | 38.23 | 28.61 | 0.37 | 12.6 | 1.66 | 0.29 | 0.07 | 1.39 | 6.73 |
| 11/02/14 | 7.78 | 13.21 | 41.62 | 30.65 | 0.20 | 18.3 | 2.13 | 0.29 | 1.40 | 0.63 | 2.63 |
| 27/02/14 | 7.69 | 13.96 | 41.35 | 30.55 | 0.07 | 17.4 | 1.81 | 0.31 | 1.52 | 0.34 | 5.76 |
| 14/03/14 | 7.86 | 14.93 | 40.68 | 30.28 | 0.14 | 15.1 | 2.19 | 0.55 | 2.32 | 0.53 | 18.10 |
| 27/03/14 | 7.88 | 13.88 | 37.91 | 28.47 | 0.10 | 19.4 | 1.48 | 0.21 | 1.28 | 0.87 | 3.51 |
| 11/04/14 | 7.99 | 17.21 | 36.52 | 27.45 | 0.06 | 19.6 | x | 0.20 | 1.11 | 0.52 | 6.13 |
| 30/04/14 | 8.03 | 18.63 | 38.03 | 27.1 | x |  | x | x | 2.09 | 1.87 | 7.97 |
| 14/05/14 | 8.05 | 19.25 | 37.68 | 28.25 | 0.17 | 17.3 | 1.43 | 0.33 | 2.70 | 0.60 | 1.23 |
| 29/05/14 | 8.02 | 16.12 | 37.26 | 28 | 0.45 | 12.9 | 1.48 | 0.27 | 3.80 | 0.79 | 8.78 |
| 11/06/14 | 7.7 | 20.84 | 27.75 | 21.43 | 0.79 | 5.5 | 0.49 | 0.11 | 2.20 | 1.70 | 15.79 |
| 30/06/14 | 8.09 | 16.49 | 36.39 | 27.13 | 0.51 | 11.8 | 1.26 | 0.09 | 1.55 | 0.50 | 6.76 |
| 11/07/14 | 8.18 | 18.47 | 38.05 | 28.52 | 0.35 | 19.3 | 1.65 | 0.09 | 2.20 | 0.87 | 9.90 |
| 25/07/14 | 8.06 | 19.61 | 36.8 | 27.15 | 0.20 | 28.7 | 1.63 | 0.15 | 3.05 | 1.22 | 10.42 |
| 20/08/14 | 7.83 | 24.55 | 38.32 | 28.12 | 0.56 | 15.1 | 1.68 | 0.19 | 3.19 | x | x |

**Table S3.** **CDOM and ancillary data of the *-holothurian* effluent water.** The a _325_values are the absorption coefficients at wavelength 325 nm (m^-1^), the S_275-295_ values are the spectral slopes at the wavelength band from 275 to 295 nm (μm^-1^), and S_R_ is the ratio of the slope between 275 and 295 nm divided by the slope between 350 and 400 nm (unitless). TDS is total dissolved solid, TOC is total organic carbon in mg C l^-1^, POM is particulate organic carbon in mgl^-1^, chl *a* is the concentration of chlorophyll a in μgl^-1^ and BA is the bacterial abundance in (x10^6^ cells ml^-1^).

| Date  (dd/mm/yy) | pH | Temp  (ºC) | Salinity  (psu) | TDS | a_325_  (m^-1^) | S_275-295_  (μm^-1^) | S_R_ | TOC  (mgC l^-1^) | POM  (mg l^-1^) | Chlorophyll *a*  (μg l^-1^) | Bacteria abundance  (x10^6^ cells ml^-1^) |
| --- | --- | --- | --- | --- | --- | --- | --- | --- | --- | --- | --- |
| 17/07/13 | 8.18 | 21.04 | 35.69 | 22.96 | 0.72 | 34.9 | 2.34 | 0.15 | x | x | 9.36 |
| 30/07/13 | 8.24 | 19.63 | 36.08 | 28.03 | x | x | x | x | x | 0.79 | x |
| 29/08/13 | 8.19 | 25.26 | 37.43 | 28.29 | 0.58 | 39.8 | 2.60 | 0.15 | x | 0.53 | 8.68 |
| 13/09/13 | 8.12 | 23.93 | 38.74 | 28.99 | 1.27 | 30.8 | 2.80 | 0.10 | x | 0.47 | 4.21 |
| 27/09/13 | 8.09 | 22.8 | 35.9 | 25.83 | 0.85 | 33.4 | 2.55 | 0.19 | 3.36 | 0.29 | 5.91 |
| 15/10/13 | 8.2 | 19.74 | 37.57 | 28.19 | 0.88 | 33.1 | 2.35 | 0.22 | 3.80 | 0.47 | 6.53 |
| 30/10/13 | 8.15 | 17.16 | 37.47 | 28.13 | 0.72 | 35.1 | 2.85 | 0.20 | 2.21 | 0.40 | 4.21 |
| 13/11/13 | 8.14 | 16.67 | 38.15 | 28.59 | 0.54 | 38.0 | 3.30 | 0.27 | 2.73 | 0.57 | 5.71 |
| 02/12/13 | 8.1 | 12.85 | 40.37 | 35.65 | 0.54 | 36.0 | 3.64 | 0.42 | 1.67 | x | 15.23 |
| 17/12/13 | 8.25 | 14.63 | 37.78 | 30.16 | 0.68 | 34.3 | 3.90 | 0.42 | 1.67 | x | 9.23 |
| 30/12/13 | 7.86 | 14.09 | 41.8 | 32.25 | x |  | x | x | 2.64 | x | 9.19 |
| 15/01/14 | 8.21 | 14.83 | 41.62 | 31.32 | x | x | x | x | 3.54 | 2.31 | 7.9.8 |
| 30/01/14 | 8.03 | 12.8 | 38.25 | 29.41 | 0.54 | 13.7 | 1.66 | 0.21 | 2.94 | 0.92 | 8.8.8 |
| 11/02/14 | 7.8 | 13.13 | 41.61 | 30.95 | 0.54 | 36.5 | 2.97 | 0.23 | 1.92 | 0.52 | 2.2.9 |
| 27/02/14 | 7.7 | 13.9 | 41.35 | 30.75 | 0.37 | 40.4 | 3.16 | 0.24 | 2.35 | x | 14.21 |
| 14/03/14 | 7.88 | 14.91 | 40.68 | 30.29 | 0.49 | 34.3 | 2.74 | 0.51 | 0.72 | 1.91 | 17.60 |
| 27/03/14 | 7.87 | 13.89 | 37.93 | 28.97 | x | x | x | x | 2.28 | 2.19 | x |
| 11/04/14 | 8.04 | 17.08 | 36.28 | 27.89 | x | x | x | x | 1.98 | x | 10.58 |
| 30/04/14 | 8.03 | 18.63 | 38.04 | 28.5 | 0.44 | 37.2 | 0.40 | 0.23 | 1.12 | 0.37 | 12.16 |
| 14/05/14 | 8.08 | 19.23 | 37.7 | 28.28 | 0.45 | 39.3 | 2.43 | 0.37 | 2.76 | 0.34 | 4.89 |
| 29/05/14 | 8.05 | 16.08 | 37.27 | 28 | 0.67 | 33.6 | 2.45 | 0.20 | 0.66 | 0.98 | 7.99 |
| 11/06/14 | 7.7 | 20.84 | 27.75 | 21.51 | 1.13 | 31.3 | 2.22 | 0.15 | 2.80 | 1.26 | 9.13 |
| 30/06/14 | 8.1 | 16.55 | 36.36 | 27.39 | 0.90 | 30.5 | 2.48 | 0.09 | 2.52 | 0.83 | 7.13 |
| 11/07/14 | 8.2 | 18.14 | 38.2 | 28.61 | 0.74 | 36.1 | 2.44 | 0.12 | 2.82 | 1.69 | 14.51 |
| 25/07/14 | 8.04 | 19.88 | 36.74 | 27.64 | 0.57 | 39.1 | 1.75 | 0.15 | 2.88 | 1.16 | 13.48 |
| 20/08/14 | 7.83 | 24.55 | 38.32 | 28.64 | x | x | x | x | x | 0.81 | x |

**Table S4. CDOM and ancillary data of the +*holothurian* tank water.** The a _325_values are the absorption coefficients at wavelength 325 nm (m^-1^), the S_275-295_ values are the spectral slopes at the wavelength band from 275 to 295 nm (μm^-1^), and S_R_ is the ratio of the slope between 275 and 295 nm divided by the slope between 350 and 400 nm (unit less). TDS is total dissolved solid, TOC is total organic carbon in mg C l^-1^, POM is particulate organic carbon in mgl^-1^, chl *a* is the concentration of chlorophyll a in μgl^-1^ and BA is the bacterial abundance in (x10^6^ cells ml^-1^).

| Date  (dd/mm/yy) | pH | Temp  (ºC) | Salinity(psu) | TDS | a_325_  (m^-1^) | S_275-295_  (μm^-1^) | S_R_ | TOC  (mg C l^-1^) | POM  (mg l^-1^) | Chlorophyll *a*  (μg l^-1^) | Bacteria abundance  (x 10^6^ cell ml^-1^) |
| --- | --- | --- | --- | --- | --- | --- | --- | --- | --- | --- | --- |
| 17/07/13 | 8.15 | 21.41 | 35.61 | 26.90 | 0.55 | 23.4 | 1.87 | 0.11 | 3.90 | x | 9.06 |
| 30/07/13 | 8.25 | 19.61 | 36.07 | 27.19 | 0.30 | 29.2 | 1.85 | 0.31 | 3.62 | 0.25 | 6.38 |
| 29/08/13 | 8.17 | 25.32 | 37.48 | 28.16 | 0.60 | 23.7 | 2.37 | 0.22 | 4.75 | 0.37 | 7.00 |
| 13/09/13 | 8.13 | 23.95 | 38.75 | 29.02 | 1.02 | 19.8 | 1.92 | 0.10 | 3.96 | 0.51 | 4.64 |
| 27/09/13 | 7.89 | 22.8 | 35.87 | 21.31 | 0.48 | 25.3 | 2.02 | 0.19 | 4.48 | 0.41 | 8.28 |
| 15/10/13 | 8.01 | 19.78 | 37.6 | 28.24 | 0.76 | 20.6 | 1.61 | 0.20 | 3.65 | 0.46 | 6.86 |
| 30/10/13 | 8.11 | 17.28 | 37.51 | 28.12 | 0.46 | 25.0 | 2.21 | 0.17 | 2.96 | 0.11 | 3.16 |
| 13/11/13 | 8.14 | 16.69 | 38.15 | 58.59 | 0.46 | 23.7 | 2.49 | 0.25 | 3.13 | 0.50 | 5.79 |
| 02/12/13 | 7.93 | 12.36 | 40.36 | 30.15 | 0.41 | 21.5 | 2.87 | 0.38 | 2.63 | 0.44 | 14.83 |
| 17/12/13 | 8.2 | 14.51 | 37.67 | 28.29 | 0.46 | 26.2 | 2.34 | 0.31 | 2.68 | 1.26 | 10.24 |
| 30/12/13 | 7.87 | 14.08 | 41.7 | 30.98 | 0.28 | 30.7 | 2.67 | 0.32 | 1.23 | 0.54 | 7.13 |
| 15/01/14 | 8.23 | 14.8 | 41.59 | 30.89 | 0.35 | 27.5 | 2.15 | 0.32 | 1.30 | 1.41 | 3.82 |
| 30/01/14 | 8.04 | 12.77 | 38.24 | 28.72 | 0.48 | 21.6 | 2.04 | 0.16 | 1.00 | 0.69 | 6.62 |
| 11/02/14 | 7.83 | 13.16 | 41.64 | 30.91 | 0.31 | 26.1 | 2.61 | 0.20 | 1.48 | 0.52 | 2.31 |
| 27/02/14 | 7.31 | 14.11 | 41.31 | 30.73 | 0.14 | 29.2 | 2.03 | 0.30 | 1.56 | 0.70 | 7.31 |
| 14/03/14 | 7.85 | 14.94 | 40.71 | 30.3 | 0.24 | 24.5 | 2.25 | 0.57 | 1.80 | 0.50 | 18.35 |
| 27/03/14 | 7.88 | 13.88 | 37.91 | 28.47 | 0.32 | 22.1 | 2.30 | 0.22 | 1.40 | 1.05 | 3.42 |
| 11/04/14 | 8.05 | 17.07 | 36.25 | 27.31 | 0.19 | 27.5 | 4.37 | 0.17 | 1.40 | 0.45 | 6.58 |
| 30/04/14 | 7.99 | 18.61 | 38.07 | 28.53 | 0.48 | 22.1 | 1.66 | 0.29 | 2.93 | 1.84 | 8.05 |
| 14/05/14 | 8.04 | 19.22 | 37.63 | 28.23 | 0.30 | 25.0 | 1.88 | 0.30 | 2.60 | 0.39 | 3.92 |
| 29/05/14 | 8.01 | 16.22 | 37.3 | 28.02 | 0.49 | 23.0 | 1.74 | 0.11 | 2.25 | 0.66 | 6.77 |
| 11/06/14 | 7.7 | 20.84 | 27.75 | 21.51 | 0.93 | 14.5 | 1.08 | 0.27 | 2.27 | 0.99 | 7.01 |
| 30/06/14 | 8.09 | 16.47 | 36.4 | 27.42 | 0.62 | 21.0 | 1.57 | 0.06 | 2.30 | 0.45 | 6.90 |
| 11/07/14 | 8.17 | 17.99 | 57.25 | 28.63 | 0.56 | 23.6 | 1.77 | 0.08 | 2.55 | 0.92 | 9.76 |
| 25/07/14 | 8.04 | 19.6 | 36.8 | 27.68 | 0.34 | 36.6 | 2.10 | 0.12 | 3.20 | 1.17 | 12.20 |
| 20/08/14 | 7.83 | 24.55 | 38.32 | 28.64 | 0.67 | 24.1 | 2.01 | 0.16 | 3.05 | 2.07 | x |

**Table S5. CDOM and ancillary data of the *-holothurian* tank water**. The a _325_values are the absorption coefficients at wavelength 325 nm (m^-1^), the S_275-295_ values are the spectral slopes at the wavelength band from 275 to 295 nm (μm^-1^), and S_R_ is the ratio of the slope between 275 and 295 nm divided by the slope between 350 and 400 nm (unit less). TDS is total dissolved solid, TOC is total organic carbon in mg C l^-1^, POM is particulate organic carbon in mgl^-1^, chl *a* is the concentration of chlorophyll a in μgl^-1^ and BA is the bacterial abundance in (x10^6^ cells ml^-1^).

| Date  (dd/mm/yy) | pH | Temp  (ºC) | Salinity  (psu) | TDS | a_325_  (m^-1^) | S_275-295_  (μm^-1^) | S_R_ | TOC  (mgC l^-1^) | POM  (mg l^-1^) | Chlorophyll *a*  (μg l^-1^) | Bacteria abundance  (x 10^6^ cells ml^-1^) |
| --- | --- | --- | --- | --- | --- | --- | --- | --- | --- | --- | --- |
| 17/07/13 | 8.18 | 21.09 | 35.68 | 26.93 | 0.79 | 26.4 | 2.18 | 0.16 | 3.72 | x | 10.22 |
| 30/07/13 | 8.23 | 19.63 | 36.08 | 27.93 | x | x | x | x | x | x | x |
| 29/08/13 | 8.18 | 25.33 | 37.43 | 28.16 | 0.90 | 26.0 | 2.17 | 0.21 | x | 2.99 | 7.60 |
| 13/09/13 | 8.14 | 23.92 | 38.80 | 29.05 | 0.82 | 27.5 | 2.20 | 0.08 | 4.44 | 0.47 | 4.20 |
| 27/09/13 | 7.96 | 22.8 | 35.92 | 25.16 | 0.76 | 27.1 | 2.19 | 0.17 | 3.40 | 0.35 | 9.30 |
| 15/10/13 | 8.16 | 19.78 | 37.62 | 28.23 | 0.93 | 24.7 | 1.93 | 0.19 | 3.46 | 0.31 | 7.35 |
| 30/10/13 | 8.14 | 17.15 | 37.47 | 28.13 | 0.77 | 26.3 | 2.68 | 0.17 | 2.32 | 0.31 | 2.80 |
| 13/11/13 | 8.14 | 16.67 | 38.17 | 28.6 | 0.62 | 29.2 | 3.04 | 0.15 | 2.77 | 0.57 | 5.84 |
| 02/12/13 | 8.11 | 12.83 | 40.36 | 35.65 | 0.62 | 26.3 | 3.81 | 0.25 | 3.42 | 1.18 | 16.93 |
| 17/12/13 | 8.26 | 14.62 | 37.77 | 28.36 | 0.75 | 29.2 | 2.56 | 0.34 | 3.12 | 1.18 | 10.06 |
| 30/12/13 | 7.86 | 14.09 | 41.8 | 32.15 | x | x | x | x | 4.14 | x | 7.13 |
| 15/01/14 | 8.22 | 14.82 | 41.61 | 31.19 | x | x | x | x | 3.60 | 2.56 | 3.88 |
| 30/01/14 | 8.03 | 12.79 | 38.24 | 29.01 | 0.56 | 29.2 | 3.32 | 0.31 | 2.82 | 0.92 | 6.66 |
| 11/02/14 | 7.85 | 13.06 | 41.63 | 30.97 | 0.56 | 30.0 | 2.65 | 0.22 | 2.28 | 0.90 | 2.31 |
| 27/02/14 | 7.66 | 13.93 | 41.34 | 30.75 | 0.44 | 32.0 | 2.60 | 0.30 | 1.97 | x | 14.11 |
| 14/03/14 | 7.88 | 14.91 | 40.6 | 30.28 | 0.54 | 26.2 | 2.50 | 0.74 | 1.54 | 2.45 | 18.34 |
| 27/03/14 | 7.87 | 13.89 | 37.92 | 28.91 | x | x | x | x | 2.34 | 3.09 | x |
| 11/04/14 | 8.04 | 17.08 | 36.26 | 27.82 | x | x | x | x | 2.04 | x | 10.59 |
| 30/04/14 | 8.03 | 18.63 | 38.05 | 28.51 | 0.52 | 29.2 | 2.68 | 0.20 | 3.12 | 0.31 | 12.07 |
| 14/05/14 | 8.08 | 19.22 | 37.69 | 28.27 | 0.52 | 30.3 | 1.99 | 0.30 | 2.34 | 0.44 | 4.73 |
| 29/05/14 | 8.04 | 16.06 | 37.28 | 28.01 | 0.82 | 25.1 | 2.07 | 0.20 | 2.28 | 0.76 | 8.08 |
| 11/06/14 | 7.7 | 20.84 | 27.75 | 21.51 | 1.16 | 22.2 | 1.79 | 0.12 | 3.30 | 1.33 | 10.02 |
| 30/06/14 | 8.1 | 16.5 | 36.37 | 27.4 | 0.93 | 22.4 | 2.11 | 0.07 | 2.58 | 0.71 | 7.19 |
| 11/07/14 | 8.2 | 18.06 | 38.21 | 28.02 | 0.81 | 26.3 | 2.17 | 0.07 | 3.12 | 1.14 | 12.15 |
| 25/07/14 | 8.04 | 19.84 | 36.75 | 27.64 | 0.62 | 32.1 | 1.51 | 0.13 | 3.28 | 1.27 | x |
| 20/08/14 | x | x | x | x | x | x | x | x | x | x | x |
